# Small but mighty: the rise of microprotein biology in neuroscience

**DOI:** 10.3389/fnmol.2024.1386219

**Published:** 2024-05-14

**Authors:** Erin E. Duffy, Elena G. Assad, Brian T. Kalish, Michael E. Greenberg

**Affiliations:** ^1^Department of Neurobiology, Harvard Medical School, Boston, MA, United States; ^2^Program in Neuroscience and Mental Health, SickKids Research Institute, Toronto, ON, Canada; ^3^Department of Molecular Genetics, University of Toronto, Toronto, ON, Canada; ^4^Division of Neonatology, Department of Paediatrics, Hospital for Sick Children, Toronto, ON, Canada

**Keywords:** microprotein, RNA translation, mitochondrial, DNA repair, mammalian, brain

## Abstract

The mammalian central nervous system coordinates a network of signaling pathways and cellular interactions, which enable a myriad of complex cognitive and physiological functions. While traditional efforts to understand the molecular basis of brain function have focused on well-characterized proteins, recent advances in high-throughput translatome profiling have revealed a staggering number of proteins translated from non-canonical open reading frames (ncORFs) such as 5′ and 3′ untranslated regions of annotated proteins, out-of-frame internal ORFs, and previously annotated non-coding RNAs. Of note, microproteins < 100 amino acids (AA) that are translated from such ncORFs have often been neglected due to computational and biochemical challenges. Thousands of putative microproteins have been identified in cell lines and tissues including the brain, with some serving critical biological functions. In this perspective, we highlight the recent discovery of microproteins in the brain and describe several hypotheses that have emerged concerning microprotein function in the developing and mature nervous system.

## Introduction

“And though she be but little, she is fierce.”

- William Shakespeare

Regulated translation of RNA into protein represents a pivotal mechanism in the control of gene expression, enabling the cell to modulate the quantity, diversity, and functionality of proteins. In the mammalian nervous system, this protein diversity allows for the establishment of specific cell types, the organization of neural circuits, and the execution of complex behaviors. Historically, one mRNA was thought to encode a single protein product, but transcriptome-wide identification of translated open reading frames (ORFs) has revealed thousands of proteins that are translated from alternative ORFs, thereby exponentially increasing proteomic diversity by encoding multiple proteins from a single mRNA. These non-canonical ORFs (ncORFs) are distinct from the coding sequence included in the reference annotation, which we will refer to as the canonical ORF. A subset of these ncORFs are microproteins, defined as proteins 100 amino acids (AA) or less in length that are translated from an independent small open reading frame (sORF, also referred to as a smORF), which have emerged as versatile regulators of cellular function. In the literature, microproteins have been interchangeably referred to as “micropeptides” and “miniproteins”, both denoting proteins that arise from sORFs. In this perspective, we will use the term “microprotein” to distinguish these proteins from proteolytic cleavage products of larger proteins.

While relatively few studies have performed rigorous functional characterization of microproteins, these small proteins have immense potential in the brain. Small secreted peptides such as Brain-Derived Neurotrophic Factor (BDNF), Nerve Growth Factor (NGF) and Neuropeptide Y (NPY) have well-established roles in neural plasticity, learning, and memory (Chao, 2003). While these neuropeptides are cleavage products from larger proteins, the *de novo* translation of sORFs may similarly serve critical cell signaling functions in the brain. Moreover, microproteins with specific functions in other tissues and cell lines, such as mitochondrial respiration, stress granule formation and DNA repair, may possess unique roles within the brain during health and disease. This perspective will highlight methods for microprotein discovery and functional characterization in the mammalian nervous system.

## Microprotein discovery in mammals

**Figure 1:**
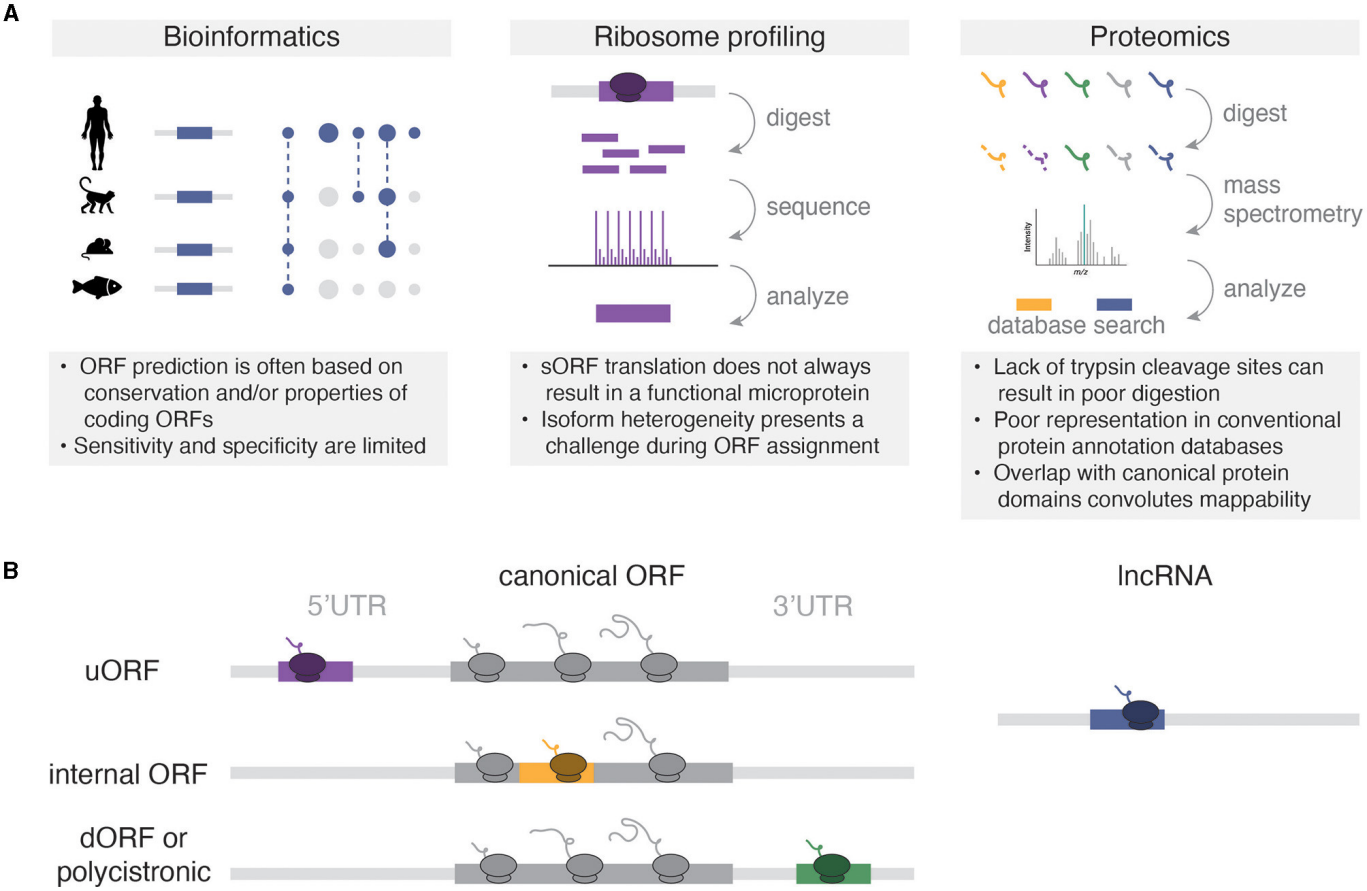
**(A)** Methods for microprotein discovery and general caveats of each approach. **(B)** Types of microRNAs relative to canonical coding sequences. Not pictured are variations of overlapping sORFs (e.g., uORFs that overlap the start codon of the canonical ORF), or rarer sORFs from non-coding RNAs like circular RNAs, pseudogenes, and microRNA precursors.

Microproteins have been historically under-studied in protein research, primarily due to the technical limitations of traditional bioinformatic and mass spectrometry analyses (Figure 1A). In bioinformatics, efforts to annotate the genome based on predicted protein-coding potential, such as those pioneered by the FANTOM consortium, introduced a cutoff of 100 AA to protein prediction to reduce the risk of false discovery of sORFs within predicted long non-coding RNAs (lncRNAs) (Okazaki et al., 2002; Dinger et al., 2008). Consequently, many potentially translated and/or functional microproteins that fell below this threshold were overlooked in the final genome annotation. Similarly, traditional mass spectrometry-based approaches have posed significant obstacles to microprotein detection due to multiple factors such as purification column size cutoffs, low microprotein abundance relative to annotated proteins, limited trypsin cleavage sites, and similarity to existing protein domains based on AA sequence (Saghatelian and Couso, 2015).

The development and widespread utilization of high throughput RNA sequencing methods to study mRNA translation subsequently enabled the discovery and cataloging of sORFs and their encoded microproteins. In particular, ribosome profiling (Ribo-seq, also known as ribosome footprinting) enabled the sequencing of ribosome-protected RNA fragments and the subsequent identification of actively translated open reading frames (Ingolia et al., 2009). This approach circumvented many technical challenges associated with proteomic discovery of microproteins and revealed >1,000 non-canonical translation events in the 5′ untranslated regions (5′UTRs) of genes in budding yeast. With the advent of Ribo-seq technologies came an explosion of studies that revealed widespread non-canonical translation across numerous eukaryotic species including zebrafish (Bazzini et al., 2014) and mouse (Harnett et al., 2022; Martinez et al., 2023), as well as human tissues including heart (van Heesch et al., 2019), kidney (Loayza-Puch et al., 2016), skeletal muscle (Wein et al., 2014), cortex (Duffy et al., 2022), and thalamus (Chothani et al., 2022). These studies also inspired targeted searches for microprotein expression using bioinformatic and mass spectrometry approaches. For example, Mackowiak et al. (2015) bioinformatically identified thousands of sORFs based on their high conservation between human, mouse, drosophila and *C. elegans*. Furthermore, modified mass spectrometry approaches that enrich small proteins and use custom protein databases generated from RNA-seq have accelerated microprotein identification (Saghatelian and Couso, 2015).

Collectively, these studies have shown that much of the transcriptome that was previously annotated as “non-coding” can encode small proteins (Figure 1B). Microproteins have been identified in 5′UTRs, where they are termed upstream open reading frames (uORFs). Classically, uORFs are thought to negatively regulate the downstream translation of canonical ORFs. For example, two uORFs in the 5′UTR of the stress response gene *Atf4* repress downstream ATF4 protein expression, and this repression is relieved by the integrated stress response (Harding et al., 2000). However, more recent high-throughput methods have shown that translational repression of downstream ORFs is uncommon for uORFs (Ingolia et al., 2009; van Heesch et al., 2019; Duffy et al., 2022), and some uORFs may exert cis- or trans-effects (Chen et al., 2020; Barragan-Iglesias et al., 2021) that depend on the sequence of the encoded microprotein rather than the act of their translation. Although downstream ORFs (dORFs) encoded by polycistronic sequences in 3′UTRs represent a relatively small proportion of all sORFs (e.g., 3.4% of sORFs in Duffy et al., 2022), these sORFs can also encode microproteins. While the mechanisms for dORF translation remain unclear, the presence of a dORF in translation reporter assays can enhance the translation of the upstream reporter ORF, suggesting a mechanistic coupling between the translation of both ORFs (Wu et al., 2020). Microproteins can also be encoded from out-of-frame sORFs with larger annotated ORFs. For example, altFUS is a highly conserved internal out-of-frame ORF translated in brain tissue, where altFUS, but not FUS, is responsible for the inhibition of autophagy in neurons (Brunet et al., 2021). Finally, many RNAs that are annotated as non-coding indeed encode functional microproteins. For example, the TUNAR lncRNA [also known as *Megamind* in zebrafish (Ulitsky et al., 2011)] encodes an evolutionarily conserved 48 AA transmembrane protein that modulates intracellular calcium dynamics through its interaction with the calcium transporter SERCA2 in the nervous system (Senís et al., 2021). These studies have revealed the translation of thousands of sORFs from annotated non-coding RNAs, thereby expanding the diversity of the known proteome.

## General properties of microproteins

Microproteins share distinct properties compared to longer annotated proteins. They are enriched for translation from non-AUG start codons (Ingolia et al., 2009; van Heesch et al., 2019; Duffy et al., 2022), and are more recently evolved on average compared to known proteins (Ruiz-Orera et al., 2014; Duffy et al., 2022; Vakirlis et al., 2022), making them challenging to detect based on sequence conservation or start codon usage alone. They also tend to exhibit lower protein expression compared to longer annotated proteins, making them more challenging to detect by mass spectrometry as discussed above. As a result, a relatively small fraction of microproteins observed as translated by Ribo-seq has subsequently been detected by mass spectrometry, sparking a debate over whether newly evolved, lowly translated or unstable microproteins have the capacity for function. These characteristics align with the classic view that evolutionarily conserved or highly abundant sORFs are more likely to carry out important functions in the cell; however, newly evolved microproteins may represent evolutionary experiments, in which a given sORF becomes translated without necessarily being conserved in subsequent evolution. While newly evolved microproteins may not have yet acquired function, it is possible for them to introduce species-specific functions to the proteome, indeed, >100 human-specific microproteins detected as translated in the human brain (Duffy et al., 2022) exhibit a significant growth phenotype when knocked out in human cell lines (Chen et al., 2020). Furthermore, several groups have found examples of newly evolved proteins that acquire function in a given species, highlighting the importance of studying these evolutionarily young proteins in addition to those that are conserved (Ruiz-Orera and Albà, 2019). In the context of neurobiology, evolutionarily new microproteins have the potential to explain some of the unique properties of the human brain relative to other species. While these hypotheses remain to be tested for human brain microproteins, they motivate the study of poorly conserved microproteins in addition to those that are highly conserved.

As protein structure is often tied to function, microproteins that adopt stable structures may also be prioritized for functional characterization. For example, microproteins that mimic the domains of larger proteins, such as Id (Benezra et al., 1990) and LITTLE ZIPPER (Wenkel et al., 2007) can act as competitive inhibitors of larger protein complexes. However, while some microproteins can adopt simple structures such as alpha helices and transmembrane domains, as a class of proteins they are enriched for intrinsically disordered regions relative to the known proteome (Duffy et al., 2022). These unique properties can confer interesting potential functions to microproteins compared to previously annotated proteins. Intrinsically disordered microproteins may be able to interact with other biomolecules either in a promiscuous or substrate-specific manner that is similar to that of intrinsically disordered regions of larger proteins, potentially allowing them to drive or disrupt macromolecular structures such as biomolecular condensates (Chakrabarti and Chakravarty, 2022). These properties make microproteins both potentially interesting and challenging to functionally characterize.

## Microprotein functional characterization

**Figure 2:**
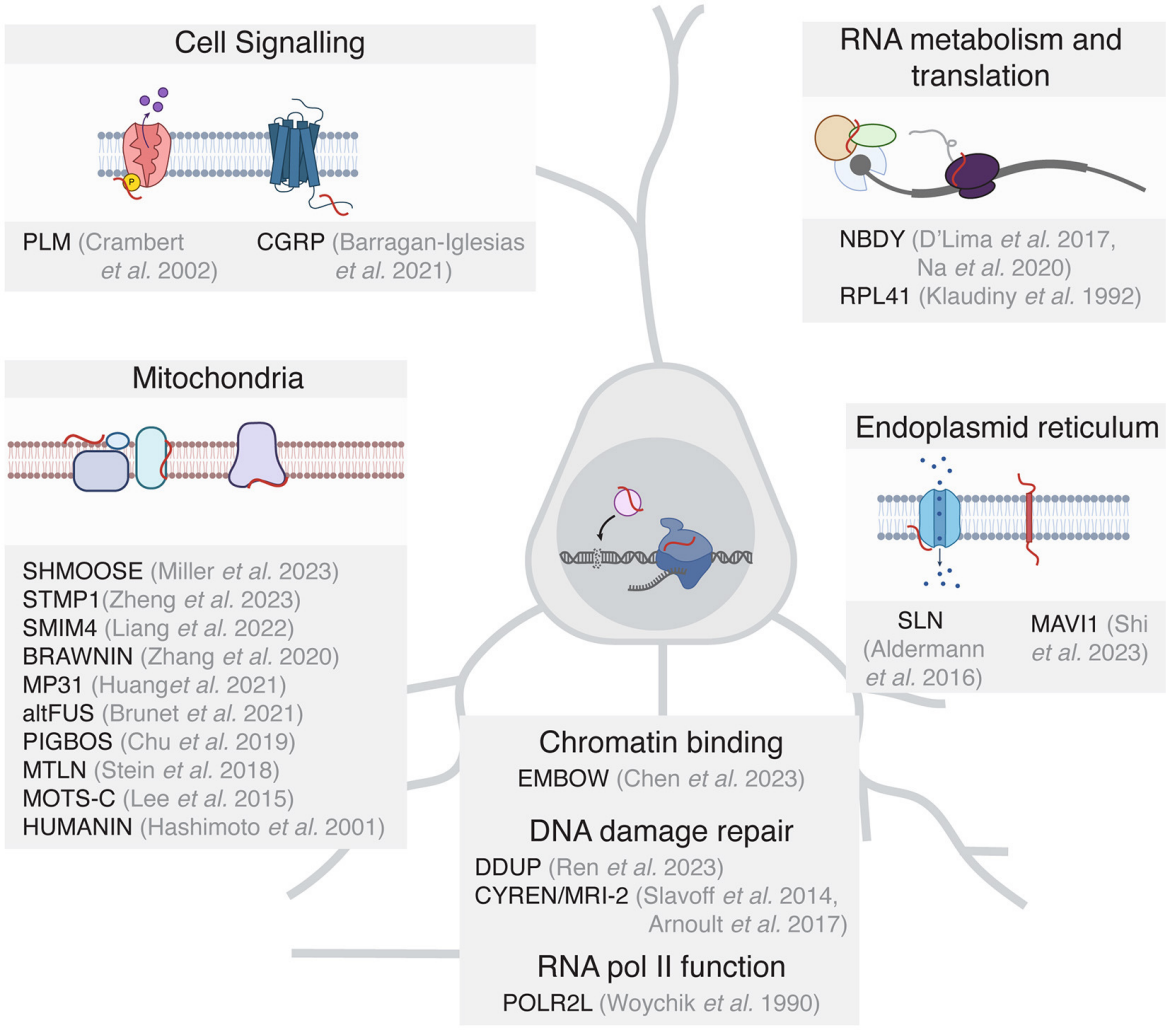
Functionally characterized microproteins grouped by functional potential in the mammalian brain. For clarity, only microproteins are included where the sORF is detected as translated in the mammalian brain.

It is important to note that the studies of microproteins in mammals are built upon excellent foundational work in non-mammalian systems (Saghatelian and Couso, 2015; Hemm et al., 2020; Kushwaha et al., 2022), and the work in non-mammalian species can inform future experiments on microproteins in the brain. While only a handful of microproteins have been functionally characterized in the nervous system to date, many microproteins in other tissues have important functions that may also be relevant in the brain. For the purposes of this perspective, we will discuss microproteins that have been functionally characterized in other tissues and reported to be expressed in the mammalian brain based on existing ribosome profiling and proteomic data (Figure 2, Wang et al., 2021; Chothani et al., 2022; Duffy et al., 2022).

Many functionally characterized microproteins have been shown to be important in mitochondrial energy homeostasis (Stein et al., 2018; Chu et al., 2019; Zhang et al., 2020; Brunet et al., 2021; Liang et al., 2022), which is critical in neurons to produce the ATP required for various neuronal processes including neurotransmitter synthesis and metabolism, maintaining ion gradients, neutralizing oxidative stress, and supporting signaling pathways. The well-characterized microprotein Humanin (HN, Hashimoto et al., 2001) can exhibit neuroprotective effects in part by binding to the cytosolic proteins Bcl2-associated X protein (BAX) and Bid to inhibit their translocation to the mitochondrial membrane. This in turn impedes Bax pore formation in the mitochondrial outer membrane and suppresses mitochondrial-dependent apoptosis (Zhu et al., 2022). In addition, several microproteins with mitochondrial function have been assayed in the mammalian brain. MP31 which is encoded by the uORF of the *PTEN* transcript, limits mitochondrial lactate-pyruvate conversion by competing with mitochondrial lactate dehydrogenase for nicotinamide adenine dinucleotide (NAD+, Huang et al., 2021). The lncRNA-encoded microprotein STMP1 is expressed in microglia and is thought to regulate mitochondrial function and protect retinal ganglion cells from oxidative damage by inhibiting the Nlrp3 inflammasome pathway (Zheng et al., 2023).

Microproteins have also been shown to play important roles in the nucleus in the context of transcription and DNA repair. The function of DNA damage repair in neurons is to preserve genomic stability and maintain the functional and structural integrity of the neuronal circuit. As neurons are post-mitotic, they rely on non-homologous end joining (NHEJ) rather than homologous repair, which requires mitotic DNA replication. While microproteins involved in nuclear function have not been characterized in neurons to date, the DDUP microprotein encoded by the DNA damage-induced lncRNA CTBP1-DT protects cells from DNA damage, likely through binding to the DNA repair factor RAD18 (Ren et al., 2023). Furthermore, the microprotein CYREN (also known as MRI-2) binds to Ku to regulate NHEJ and double-stranded break repair (Slavoff et al., 2014; Arnoult et al., 2017). Other microproteins function as subunits of RNA polymerase II (POLR2L, Woychik and Young, 1990) and regulate the binding of transcription factors to chromatin. One such protein is the microprotein EMBOW, which facilitates WDR5 protein complex assembly and regulates the DNA binding specificity of the complex (Chen et al., 2023). As WDR5 also regulates neurodevelopment and dendritic polarity (Ka et al., 2022), it is plausible that microproteins such as EMBOW participate in the regulation of transcription during nervous system development.

Several microproteins are themselves transmembrane proteins or interact with proteins on cellular membranes and facilitate cell signaling. For example, the microprotein phospholemman (PLM) is a single-pass transmembrane protein that regulates the activity of the Na,K-ATPase (NK) complex to maintain Na+ and K+ gradients across cell membranes (Crambert et al., 2002). The microprotein CGRP, which is expressed from a uORF of the calcitonin (*Calca*) gene, promotes pain sensitization in mouse dorsal root ganglia through GPCR signaling (Barragan-Iglesias et al., 2021). Several SERCA-inhibiting microproteins regulate calcium signaling in the heart (Anderson et al., 2016), and one of these microproteins, SLN, is also translated in the human brain (Duffy et al., 2022), suggesting a potentially interesting role in neuronal calcium signaling. The microprotein MAVI1, encoded by the gene *Smim30*, is a transmembrane protein localized to the endoplasmic reticulum where it interacts with the mitochondrial protein MAVS to block innate immune responses (Shi et al., 2023). The expression of MAVI1 in the human brain suggests potential additional functions of MAVI1 beyond antiviral innate immune responses.

Finally, there are limited but interesting examples of microproteins that regulate RNA metabolism and translational control. The 25 AA ribosomal subunit RPL41 is a highly conserved microprotein from yeast to mammals (Klaudiny et al., 1992). RPL41 expression has recently been suggested to be a useful biomarker for Alzheimer's disease (Cruz-Rivera et al., 2018). The microprotein NoBody (NBDY) regulates mRNA decapping and stability through its interaction with processing bodies, cytoplasmic ribonucleoprotein (RNP) granules that are made up of translationally repressed mRNAs and proteins related to mRNA decay (D'Lima et al., 2017), where P-bodies are hypothesized to regulate local RNA translation at synapses (Zeitelhofer et al., 2008). Investigating the role that microproteins play in RNA translation and metabolism in neurons represents a fascinating future direction in microprotein research.

## Discussion

### Challenges of studying microproteins

The precise spatiotemporal expression of proteins is fundamental to synapse plasticity and circuit remodeling. Much of the work to date on the role of translation in the nervous system has focused on the canonical proteome, but advances in proteomics and genomics in the last decade have revealed an expansive landscape of ncORFs, including sORFs that encode microproteins. Moving forward, the noncanonical proteome is a potentially rich source of underexplored neurobiology, but several challenges have limited mechanistic studies. Herein, we define critical scientific priorities, technical challenges, and potential opportunities for investigation that lie at the intersection of microprotein biology and neuroscience.

The foremost challenge is identifying a high-confidence set of brain microproteins, which can then be exploited for functional interrogation. There is currently a lack of standardization in the experimental methods, data quality control, and analysis of sORFs and microproteins, which has led to significant variability in the identification of translated sORFs. Given the need to adopt rigorous, uniform standards for microprotein validation, several groups have proposed consensus definitions to improve the reliability and consistency of ncORF and protein coding identification (Mudge et al., 2022; Chothani et al., 2023; Prensner et al., 2023). These definitions include the independent identification of a sORF across multiple studies, detection by multiple experimental methods (e.g., Ribo-seq plus mass spectrometry, epitope tagging and western blot, or detection by endogenous antibodies), and/or the presence within the microprotein of disease-associated mutations (Table 1).

**Table 1:** Suggested criteria for prioritizing sORFs for functional characterization.

**Criteria**	**Comments**
Detection by more than one experimental method (e.g., Ribo-seq plus mass spectrometry, epitope tagging and western blot, or detection by endogenous antibodies)	Proteins that are expressed at high enough levels to be detected by mass spectrometry or western blot are more likely to execute important functions
Evolutionary conservation	While not required for function, selective pressure increases the probability of function
Homology with protein of known function	Microproteins that mimic known proteins can act as positive or negative regulators of cellular functions
Presence of disease-associated mutations	This includes microproteins whose expression is misregulated in disease states

Importantly, not all criteria must be simultaneously satisfied.

Another challenge for microprotein neurobiology is the difficulty in prioritizing candidate sORFs for functional investigation. Approaches to filter and prioritize sORFs, based on their physicochemical properties, sequence conservation, predicted structure (using AlphaFold) and subcellular localization are likely to accelerate biological insight. However, these approaches have significant limitations when applied to microproteins. AlphaFold, for instance, has not been trained on microproteins and thus may provide misleading predictions for putative microproteins and their potential protein-protein interactions (Jumper et al., 2021). Empirical data will be necessary to train more comprehensive machine-learning models for noncanonical proteins. Another potential avenue to elucidate functionally relevant microproteins in the brain is to identify candidates that are associated with neurologic disease vulnerability. Specifically, sORFs with enrichment of disease-associated genomic variants may be more likely to have biologically relevant functions. For example, single nucleotide polymorphisms (SNPs) in patients with Alzheimer's disease have been identified in the mitochondrial microproteins HN and SCHMOOSE (Niikura, 2022; Miller et al., 2023). However, such analyses are complicated by the proximity or overlap of sORFs with canonical ORFs and therefore require the development of new computational tools to incorporate non-canonical ORFs into genome annotations and variant calling algorithms. Alternatively, microproteins that show differential expression in different neurodevelopmental or disease states offer interesting candidates for functional characterization. For example, thousands of microproteins detected in the human brain show differential RNA expression and translatability in the fetal vs. adult brain (Duffy et al., 2022).

To circumvent the laborious process of functionally characterizing individual microproteins, several groups have pioneered high-throughput, unbiased testing of microprotein function. For example, Chen et al. (2020) used CRISPR-Cas9 strategies to investigate the function of thousands of microproteins in mammalian cells by mutating the start codon of individual sORFs and identified hundreds of microproteins that are important for cell growth and fitness. Hofman et al. (2024) used a similar approach to identify microproteins translated from uORFs and lncRNAs that are required for medullablastoma cell survival. Conversely, a recently described translation-activating RNA technology may be a useful technique to promote the targeted upregulation of specific sORFs (Cao et al., 2023). While these approaches facilitate the nomination of biologically important microproteins from the thousands of potential sORF candidates, they have, to date, been limited to biological assays of cell growth and survival. Future screens will need to employ more neurobiologically relevant assays, including neural differentiation, electrophysiology, bioenergetics, and synapse complexity and composition.

Beyond the need to confidently identify, prioritize, and predict functionality of brain ncORFs and microproteins, the field will require new computational and experimental tools to interrogate microprotein function at single-cell resolution in the brain. Microprotein expression in the brain may be cell type-specific, developmentally regulated, or expressed in response to specific stimuli or disease states, all of which will be challenging to study using current methods and may require a combination of *in vitro* models and an examination of primary tissue. Recently described approaches for single-cell ribosome profiling (Ozadam et al., 2023) and *in situ* spatial translatome mapping (Zeng et al., 2023) raise the promise of studying translation more precisely in heterogeneous tissues such as the brain. For example, microglia may employ a unique repertoire of microproteins, as immune cells often leverage microproteins in the context of antigen recognition and presentation (Malekos and Carpenter, 2022). Therefore, ribosome profiling of specific glial populations, combined with proteomics approaches to identify small immunopeptides presented on the cell surface, are likely to uncover unique microproteins that contribute to the neuro-immune landscape.

### Future directions and conclusions

Moving forward, the brain poses unique challenges to microprotein research that will require the development and consensus of rigorous experimental and computational approaches to define and characterize microproteins across development and disease. Despite these challenges, microproteins remain an exciting avenue for future research aimed at understanding the importance of non-canonical translation for cognitive development and brain function.

## Data availability statement

The original contributions presented in the study are included in the article/supplementary material, further inquiries can be directed to the corresponding authors.

## Author contributions

ED: Writing—original draft, Writing—review & editing. EA: Writing—review & editing. BK: Writing—original draft, Writing—review & editing. MG: Writing—review & editing.

## References

[B1] Anderson D. M. Makarewich C. A. Anderson K. M. Shelton J. M. Bezprozvannaya S. Bassel-Duby R.. (2016). Widespread control of calcium signaling by a family of SERCA-inhibiting micropeptides. Sci. Signal 9:ra119. 10.1126/scisignal.aaj1460 27923914 PMC5696797

[B2] Arnoult N. Correia A. Ma J. Merlo A. Garcia-Gomez S. Maric M.. (2017). Regulation of DNA repair pathway choice in S and G2 phases by the NHEJ inhibitor CYREN. Nature 549, 548–552. 10.1038/nature24023 28959974 PMC5624508

[B3] Barragan-Iglesias P. Kunder N. Wanghzou A. Black B. Ray P. R. Lou T.-F.. (2021). A peptide encoded within a 5′ untranslated region promotes pain sensitization in mice. Pain 162, 1864–1875. 10.1097/j.pain.0000000000002191 33449506 PMC8119312

[B4] Bazzini A. A. Johnstone T. G. Christiano R. Mackowiak S. D. Obermayer B. Fleming E. S.. (2014). Identification of small ORFs in vertebrates using ribosome footprinting and evolutionary conservation. EMBO J. 33, 981–993. 10.1002/embj.201488411 24705786 PMC4193932

[B5] Benezra R. Davis R. L. Lockshon D. Turner D. L. Weintraub H. (1990). The protein Id: a negative regulator of helix-loop-helix DNA binding proteins. Cell 61, 49–59. 10.1016/0092-8674(90)90214-y 2156629

[B6] Brunet M. A. Jacques J.-F. Nassari S. Tyzack G. E. McGoldrick P. Zinman L.. (2021). The FUS gene is dual-coding with both proteins contributing to FUS-mediated toxicity. EMBO Rep. 22:e50640. 10.15252/embr.202050640 33226175 PMC7788448

[B7] Cao Y. Liu H. Lu S. S. Jones K. A. Govind A. P. Jeyifous O.. (2023). RNA-based translation activators for targeted gene upregulation. Nat. Commun. 14:6827. 10.1038/s41467-023-42252-z 37884512 PMC10603104

[B8] Chakrabarti P. Chakravarty D. (2022). Intrinsically disordered proteins/regions and insight into their biomolecular interactions. Biophys. Chem. 283:106769. 10.1016/j.bpc.2022.106769 35139468

[B9] Chao M. V. (2003). Neurotrophins and their receptors: a convergence point for many signalling pathways. Nat. Rev. Neurosci. 4, 299–309. 10.1038/nrn1078 12671646

[B10] Chen J. Brunner A.-D. Cogan J. Z. Nuñez J. K. Fields A. P. Adamson B.. (2020). Pervasive functional translation of noncanonical human open reading frames. Science 367, 1140–1146. 10.1126/science.aay0262 32139545 PMC7289059

[B11] Chen Y. Su H. Zhao J. Na Z. Jiang K. Bacchiocchi A.. (2023). Unannotated microprotein EMBOW regulates the interactome and chromatin and mitotic functions of WDR5. Cell Rep. 42:113145. 10.1016/j.celrep.2023.113145 37725512 PMC10629662

[B12] Chothani S. Ho L. Schafer S. Rackham O. (2023). Discovering microproteins: making the most of ribosome profiling data. RNA Biol. 20, 943–954. 10.1080/15476286.2023.2279845 38013207 PMC10730196

[B13] Chothani S. P. Adami E. Widjaja A. A. Langley S. R. Viswanathan S. Pua C. J.. (2022). A high-resolution map of human RNA translation. Mol. Cell 82, 2885–2899.e8. 10.1016/j.molcel.2022.06.023 35841888

[B14] Chu Q. Martinez T. F. Novak S. W. Donaldson C. J. Tan D. Vaughan J. M.. (2019). Regulation of the ER stress response by a mitochondrial microprotein. Nat. Commun. 10:4883. 10.1038/s41467-019-12816-z 31653868 PMC6814811

[B15] Crambert G. Fuzesi M. Garty H. Karlish S. Geering K. (2002). Phospholemman (FXYD1) associates with Na,K-ATPase and regulates its transport properties. Proc. Natl. Acad. Sci. USA. 99, 11476–11481. 10.1073/pnas.182267299 12169672 PMC123281

[B16] Cruz-Rivera Y. E. Perez-Morales J. Santiago Y. M. Gonzalez V. M. Morales L. Cabrera-Rios M.. (2018). A selection of important genes and their correlated behavior in Alzheimer's disease. J. Alzheimers. Dis. 65, 193–205. 10.3233/JAD-170799 30040709 PMC6087431

[B17] Dinger M. E. Pang K. C. Mercer T. R. Mattick J. S. (2008). Differentiating protein-coding and noncoding RNA: challenges and ambiguities. PLoS Comput. Biol. 4:e1000176. 10.1371/journal.pcbi.1000176 19043537 PMC2518207

[B18] D'Lima N. G. Ma J. Winkler L. Chu Q. Loh K. H. Corpuz E. O.. (2017). A human microprotein that interacts with the mRNA decapping complex. Nat. Chem. Biol. 13, 174–180. 10.1038/nchembio.2249 27918561 PMC5247292

[B19] Duffy E. E. Finander B. Choi G. Carter A. C. Pritisanac I. Alam A.. (2022). Developmental dynamics of RNA translation in the human brain. Nat. Neurosci. 25, 1353–1365. 10.1038/s41593-022-01164-9 36171426 PMC10198132

[B20] Harding H. P. Novoa I. Zhang Y. Zeng H. Wek R. Schapira M.. (2000). Regulated translation initiation controls stress-induced gene expression in mammalian cells. Mol. Cell 6, 1099–1108. 10.1016/s1097-2765(00)00108-8 11106749

[B21] Harnett D. Ambrozkiewicz M. C. Zinnall U. Rusanova A. Borisova E. Drescher A. N.. (2022). A critical period of translational control during brain development at codon resolution. Nat. Struct. Mol. Biol. 29, 1277–1290. 10.1038/s41594-022-00882-9 36482253 PMC9758057

[B22] Hashimoto Y. Ito Y. Niikura T. Shao Z. Hata M. Oyama F.. (2001). Mechanisms of neuroprotection by a novel rescue factor humanin from Swedish mutant amyloid precursor protein. Biochem. Biophys. Res. Commun. 283, 460–468. 10.1006/bbrc.2001.4765 11327724

[B23] Hemm M. R. Weaver J. Storz G. (2020). Escherichia coli small proteome. EcoSal Plus 2019:9. 10.1128/ecosalplus.ESP-0031-2019 32385980 PMC7212919

[B24] Hofman D. A. Ruiz-Orera J. Yannuzzi I. Murugesan R. Brown A. Clauser K. R.. (2024). Translation of non-canonical open reading frames as a cancer cell survival mechanism in childhood medulloblastoma. Mol Cell 84, 261–276.e18. 10.1016/j.molcel.2023.12.003 38176414 PMC10872554

[B25] Huang N. Li F. Zhang M. Zhou H. Chen Z. Ma X.. (2021). An upstream open reading frame in phosphatase and tensin homolog encodes a circuit breaker of lactate metabolism. Cell Metab 33, 128–144.e9. 10.1016/j.cmet.2020.12.008 33406399

[B26] Ingolia N. T. Ghaemmaghami S. Newman J. R. S. Weissman J. S. (2009). Genome-wide analysis *in vivo* of translation with nucleotide resolution using ribosome profiling. Science. 324, 218–223. 10.1126/science.1168978 19213877 PMC2746483

[B27] Jumper J. Evans R. Pritzel A. Green T. Figurnov M. Ronneberger O.. (2021). Highly accurate protein structure prediction with AlphaFold. Nature 596, 583–589. 10.1038/s41586-021-03819-2 34265844 PMC8371605

[B28] Ka M. Kim H.-G. Kim W.-Y. (2022). WDR5-HOTTIP histone modifying complex regulates neural migration and dendrite polarity of pyramidal neurons via reelin signaling. Mol. Neurobiol. 59, 5104–5120. 10.1007/s12035-022-02905-4 35672601 PMC9378496

[B29] Klaudiny J. von der Kammer H. Scheit K. H. (1992). Characterization by cDNA cloning of the mRNA of a highly basic human protein homologous to the yeast ribosomal protein YL41. Biochem. Biophys. Res. Commun. 187, 901–906. 10.1016/0006-291x(92)91282-u 1326959

[B30] Kushwaha A. K. Dwivedi S. Mukherjee A. Lingwan M. Dar M. A. Bhagavatula L.. (2022). Plant microProteins: small but powerful modulators of plant development. iScience 25, 105400. 10.1016/j.isci.2022.105400 36353725 PMC9638782

[B31] Liang C. Zhang S. Robinson D. Ploeg M. V. Wilson R. Nah J.. (2022). Mitochondrial microproteins link metabolic cues to respiratory chain biogenesis. Cell Rep. 40, 111204. 10.1016/j.celrep.2022.111204 35977508

[B32] Loayza-Puch F. Rooijers K. Buil L. C. M. Zijlstra J. Oude Vrielink J. F. Lopes R.. (2016). Tumour-specific proline vulnerability uncovered by differential ribosome codon reading. Nature 530, 490–494. 10.1038/nature16982 26878238

[B33] Mackowiak S. D. Zauber H. Bielow C. Thiel D. Kutz K. Calviello L.. (2015). Extensive identification and analysis of conserved small ORFs in animals. Genome Biol. 16, 179. 10.1186/s13059-015-0742-x 26364619 PMC4568590

[B34] Malekos E. Carpenter S. (2022). Short open reading frame genes in innate immunity: from discovery to characterization. Trends Immunol. 43, 741–756. 10.1016/j.it.2022.07.005 35965152 PMC10118063

[B35] Martinez T. F. Lyons-Abbott S. Bookout A. L. De Souza E. V. Donaldson C. Vaughan J. M.. (2023). Profiling mouse brown and white adipocytes to identify metabolically relevant small ORFs and functional microproteins. Cell Metab. 35, 166–183.e11. 10.1016/j.cmet.2022.12.004 36599300 PMC9889109

[B36] Miller B. Kim S.-J. Mehta H. H. Cao K. Kumagai H. Thumaty N.. (2023). Mitochondrial DNA variation in Alzheimer's disease reveals a unique microprotein called SHMOOSE. Mol. Psychiatry 28, 1813–1826. 10.1038/s41380-022-01769-3 36127429 PMC10027624

[B37] Mudge J. M. Ruiz-Orera J. Prensner J. R. Brunet M. A. Calvet F. Jungreis I.. (2022). Standardized annotation of translated open reading frames. Nat. Biotechnol. 40, 994–999. 10.1038/s41587-022-01369-0 35831657 PMC9757701

[B38] Niikura T. (2022). Humanin and Alzheimer's disease: the beginning of a new field. Biochim. Biophys. Acta Gen Subj. 1866:130024. 10.1016/j.bbagen.2021.130024 34626746

[B39] Okazaki Y. Furuno M. Kasukawa T. Adachi J. Bono H. Kondo S.. (2002). Analysis of the mouse transcriptome based on functional annotation of 60,770 full-length cDNAs. Nature 420, 563–573. 10.1038/nature01266 12466851

[B40] Ozadam H. Tonn T. Han C. M. Segura A. Hoskins I. Rao S.. (2023). Single-cell quantification of ribosome occupancy in early mouse development. Nature 618, 1057–1064. 10.1038/s41586-023-06228-9 37344592 PMC10307641

[B41] Prensner J. R. Abelin J. G. Kok L. W. Clauser K. R. Mudge J. M. Ruiz-Orera J.. (2023). What can Ribo-seq, immunopeptidomics, and proteomics tell us about the noncanonical proteome? Mol. Cell. Proteomics 22, 100631. 10.1016/j.mcpro.2023.100631 37572790 PMC10506109

[B42] Ren L. Qing X. Wei J. Mo H. Liu Y. Zhi Y.. (2023). The DDUP protein encoded by the DNA damage-induced CTBP1-DT lncRNA confers cisplatin resistance in ovarian cancer. Cell Death Dis. 14, 568. 10.1038/s41419-023-06084-5 37633920 PMC10460428

[B43] Ruiz-Orera J. Albà M. M. (2019). Translation of small open reading frames: roles in regulation and evolutionary innovation. Trends Genet. 35, 186–198. 10.1016/j.tig.2018.12.003 30606460

[B44] Ruiz-Orera J. Messeguer X. Subirana J. A. Alba M. M. (2014). Long non-coding RNAs as a source of new peptides. Elife 3:e03523. 10.7554/eLife.03523 25233276 PMC4359382

[B45] Saghatelian A. Couso J. P. (2015). Discovery and characterization of smORF-encoded bioactive polypeptides. Nat. Chem. Biol. 11, 909–916. 10.1038/nchembio.1964 26575237 PMC4956473

[B46] Senís E. Esgleas M. Najas S. Jiménez-Sábado V. Bertani C. Giménez-Alejandre M.. (2021). TUNAR lncRNA encodes a microprotein that regulates neural differentiation and neurite formation by modulating calcium dynamics. Front. Cell Dev. Biol. 9, 747667. 10.3389/fcell.2021.747667 35036403 PMC8758570

[B47] Shi T.-T. Huang Y. Li Y. Dai X.-L. He Y.-H. Ding J.-C.. (2023). MAVI1, an endoplasmic reticulum-localized microprotein, suppresses antiviral innate immune response by targeting MAVS on mitochondrion. Sci. Adv. 9:eadg7053. 10.1126/sciadv.adg7053 37656786 PMC10854431

[B48] Slavoff S. A. Heo J. Budnik B. A. Hanakahi L. A. Saghatelian A. (2014). A human short open reading frame (sORF)-encoded polypeptide that stimulates DNA end joining. J. Biol. Chem. 289, 10950–10957. 10.1074/jbc.C113.533968 24610814 PMC4036235

[B49] Stein C. S. Jadiya P. Zhang X. McLendon J. M. Abouassaly G. M. Witmer N. H.. (2018). Mitoregulin: a lncRNA-encoded microprotein that supports mitochondrial supercomplexes and respiratory efficiency. Cell Rep. 23, 3710–3720.e8. 10.1016/j.celrep.2018.06.002 29949756 PMC6091870

[B50] Ulitsky I. Shkumatava A. Jan C. H. Sive H. Bartel D. P. (2011). Conserved function of lincRNAs in vertebrate embryonic development despite rapid sequence evolution. Cell 147, 1537–1550. 10.1016/j.cell.2011.11.055 22196729 PMC3376356

[B51] Vakirlis N. Vance Z. Duggan K. M. McLysaght A. (2022). De novo birth of functional microproteins in the human lineage. Cell Rep. 41:111808. 10.1016/j.celrep.2022.111808 36543139 PMC10073203

[B52] van Heesch S. Witte F. Schneider-Lunitz V. Schulz J. F. Adami E. Faber A. B.. (2019). The translational landscape of the human heart. Cell 178, 242–260.e29. 10.1016/j.cell.2019.05.010 31155234

[B53] Wang H. Wang Y. Yang J. Zhao Q. Tang N. Chen C.. (2021). Tissue- and stage-specific landscape of the mouse translatome. Nucleic Acids Res. 49, 6165–6180. 10.1093/nar/gkab482 34107020 PMC8216458

[B54] Wein N. Vulin A. Falzarano M. S. Szigyarto C. A.-K. Maiti B. Findlay A.. (2014). Translation from a DMD exon 5 IRES results in a functional dystrophin isoform that attenuates dystrophinopathy in humans and mice. Nat. Med. 20, 992–1000. 10.1038/nm.3628 25108525 PMC4165597

[B55] Wenkel S. Emery J. Hou B.-H. Evans M. M. S. Barton M. K. (2007). A feedback regulatory module formed by LITTLE ZIPPER and HD-ZIPIII genes. Plant Cell 19, 3379–3390. 10.1105/tpc.107.055772 18055602 PMC2174893

[B56] Woychik N. A. Young R. A. (1990). RNA polymerase II subunit RPB10 is essential for yeast cell viability. J. Biol. Chem. 265, 17816–17819. 2211663

[B57] Wu Q. Wright M. Gogol M. M. Bradford W. D. Zhang N. Bazzini A. A. (2020). Translation of small downstream ORFs enhances translation of canonical main open reading frames. EMBO J. 39:e104763. 10.15252/embj.2020104763 32744758 PMC7459409

[B58] Zeitelhofer M. Macchi P. Dahm R. (2008). Perplexing bodies: the putative roles of P-bodies in neurons. RNA Biol. 5, 244–248. 10.4161/rna.6948 19182533

[B59] Zeng H. Huang J. Ren J. Wang C. K. Tang Z. Zhou H.. (2023). Spatially resolved single-cell translatomics at molecular resolution. Science 380:eadd3067. 10.1126/science.add3067 37384709 PMC11146668

[B60] Zhang S. Reljić B. Liang C. Kerouanton B. Francisco J. C. Peh J. H.. (2020). Mitochondrial peptide BRAWNIN is essential for vertebrate respiratory complex III assembly. Nat. Commun. 11:1312. 10.1038/s41467-020-14999-2 32161263 PMC7066179

[B61] Zheng X. Wang M. Liu S. Chen H. Li Y. Yuan F.. (2023). A lncRNA-encoded mitochondrial micropeptide exacerbates microglia-mediated neuroinflammation in retinal ischemia/reperfusion injury. Cell Death Dis. 14:126. 10.1038/s41419-023-05617-2 36792584 PMC9932084

[B62] Zhu S. Hu X. Bennett S. Xu J. Mai Y. (2022). The molecular structure and role of humanin in neural and skeletal diseases, and in tissue regeneration. Front. Cell Dev. Biol. 10:823354. 10.3389/fcell.2022.823354 35372353 PMC8965846

